# The Shared Book Reading Corpus: An audiovisual collection of 44 multimodal caregiver-infant interactions

**DOI:** 10.1038/s41597-025-05894-w

**Published:** 2025-09-30

**Authors:** Teruni Ahamat, Jiahao Yang, Sotaro Kita, Suzanne Aussems

**Affiliations:** 1 Department of Psychology, University of Warwick, Coventry, UK; 2 Institute for Digital Security and Behaviour, University of Bath, Bath, UK

**Keywords:** Human behaviour, Databases

## Abstract

The Shared Book Reading Corpus is a collection of audiovisual recordings of English-speaking caregivers and 13–14-month-old infants (*N* = 44 dyads) reading together in a controlled lab setting. Caregivers were instructed to read a *First 100 Words* picture-book with their infant as they naturally would at home. An overview camera recorded the full interaction, while two head-mounted cameras worn by all caregivers and most infants captured their individual perspectives. The corpus also contains detailed caregiver speech transcriptions, comprehensive family demographic and socioeconomic information for all dyads, as well as measures of all infants’ vocabulary and pointing gesture development. The corpus is documented on Databrary and provides a valuable resource for investigating rich multimodal caregiver-infant interactions in early development.

## Background & Summary

Infants develop in rich multimodal environments. Their socio-emotional, cognitive, and linguistic faculties are shaped by dynamic social interactions with their caregivers^1–6^. In these interactions, both infants and caregivers produce multimodal behaviours such as gestures, eye gaze, facial expressions, and intonation. These multimodal behaviours enhance meaning-making in communication^7,8^ and therefore play a crucial role in learning and development. A robust body of research over the past several decades has highlighted the developmental significance of such multimodal behaviours. Infants actively shape their development through behaviours such as gesture^9,10^ and eye gaze^11^. Likewise, caregivers and other adults influence infants’ development using multimodal behaviours, including gesture^10,12,13^, eye gaze^14–16^, facial expressions^17–19^, and prosody^20–22^. Moreover, emerging theoretical frameworks that situate infants’ development within real-world contexts have further highlighted these influences^23,24^. The current paper describes a collection of audiovisual recordings, which captures rich multimodal caregiver-infant interactions during shared book reading.

There is a call for developing and sharing collections of audiovisual data that capture the inherently multimodal nature of caregiver-infant interactions^25^. Collecting and documenting this type of data demands significant time and resources. In addition, ethical concerns around privacy, storage, and management have historically made open sharing of audiovisual data challenging. However, advances in open science practices and data sharing policies now allow for the secure sharing of audiovisual data through online repositories. For example, Databrary, a web-based library of audiovisual data, provides safe access to audiovisual recordings to authorised researchers^26^. As a result, new multimodal corpora are now being curated and shared on this platform. For example, Tamis-Lemonda and Adolph’s^27^ corpus includes audiovisual recordings of semi-structured and free play interactions between infants and caregivers in the family home. This corpus has been instrumental in validating the embodied learning hypothesis, demonstrating that infants form object-label mappings through everyday interactions with objects during social play^28^ and that infants’ physical actions are closely linked to the verbs caregivers use in the home^29^. Similarly, Sullivan and colleagues’^30^ SAYCam longitudinal corpus on Databrary consists of everyday interactions at home, in neighbourhoods, and workplaces, recorded from the infants’ perspective (via a Veho head-mounted camera on a custom mounting headband). This corpus has been used to examine how infants’ visual experiences support early word learning, showing that exposure to naturalistic, infant-centric data enables neural networks to form word-referent mappings, develop broad semantic categories, and align visual and linguistic systems^31,32^. Most recently, Gu *et al*.’s^33^ ECOLANG corpus captures semi-naturalistic conversations between 3- to 4-year-olds and adults in the family home about objects that are present or absent. This corpus provides valuable insights into how multimodal behaviours are used in caregiver-child communication, beyond the here-and-now, supporting multimodal language acquisition research^34^.

Shared book reading is an important interactive activity that promotes infants’ cognitive^35,36^, socioemotional^37,38^ and language development^39–44^. This routine practice encourages meaningful interactions between caregivers and infants. Corpus studies on shared book reading have examined how caregivers’ speech supports infants’ language development^45–47^ and how variations in book format influence the quality of interaction^48^. Additionally, a small number of studies have adopted a multimodal approach, investigating how caregiver behaviours—such as gesture and eye gaze—create word-learning opportunities and aid word-object mapping^49,50^. While some corpora documenting shared book reading interactions are accessible online, these are limited to audio recordings and transcripts^46^. To our knowledge, there is currently no accessible audiovisual collection focused on shared book reading between caregivers and infants.

Our Shared Book Reading Corpus^51^ offers unique multimodal contributions. For instance, it captures shared book reading interactions from both third- and first-person perspectives. In the third-person perspective, an overview camera provides a frontal view of the caregiver and infant sat in a reading chair. The corpus also contains two first-person perspectives for all caregivers and most infants. In the caregiver first-person perspective, a head-mounted camera captures caregiver’s perspective during reading which often included the infant and the book. In the infant first-person perspective, a head-mounted camera captures the infant’s perspective during reading which often included the book and the caregiver’s hands. Head-mounted cameras were used to provide unique insights into caregivers’ and infants’ real-world visual experiences^1,52,53^. Together, these recordings from three perspectives capture facial expressions and body movements, while also providing a clear view of the book pages and hand movements and hand gestures from both the caregivers’ and infants’ perspectives. Additionally, the high-quality microphone captures the intricate acoustic features of their verbal communication and vocalisations. Time-stamped transcripts of the caregivers’ speech during the reading session are also part of the corpus. This corpus therefore enables a fine-grained analysis of the multimodal features of shared book reading interactions. Moreover, the corpus permits further examination of the impact of book features—such as illustration richness, text layout, realistic images, and minimal-text—on early reading interactions^48,54,55^. In addition, it enables analysis of infant-centric and caregiver-centric multimodal input during early social interactions^1,52,53,56–58^. It may also be possible to use the corpus akin to SAYCam^30^ to train language models on infant-centric input, thereby generating humanlike learning machines^31,32^. Furthermore, researchers could apply computer vision techniques to the infants’ head-mounted camera data to investigate visual aspects of the input infants receive during shared book reading and how this shapes their learning^59^. The nature of the audiovisual recordings thus offers wide range of possibilities for further analysis but is especially well-designed for multimodal analysis.

Finally, the corpus also contains demographic and socioeconomic data, and caregiver-reported measures of infants’ vocabulary and pointing gesture development. Researchers may wish to include certain demographic and socioeconomic variables in their analysis or use them to describe the sample characteristics and evaluate the generalisability of any study findings when reporting research with infants^60^. The corpus also includes caregiver-reported assessments of infants’ vocabulary development, collected using the UK Communicative Development Inventory^61^ at the time of the reading session, as well as data on infants’ pointing development, measured via the QPOINT^62^. Thus, the combination of observational data from the audiovisual recordings, and collection of parent-report measures, may aid researchers in analysing infants’ development. For example, researchers can use the multimodal resource to validate and further investigate the role of behaviours such as gesture, facial expressions, eye gaze and prosody in infants’ language development^13,63–66^, nonverbal communicative development^10,49,67,68^, joint engagement and attention^6,69,70^, and socio-pragmatic development^71–73^. Overall, the caregiver-infant Shared Book Reading Corpus^51^ provides a comprehensive collection of multimodal interactions between caregivers and infants during shared book reading.

This paper reports the development of the corpus, describing the ethical and data collection protocols, data processing pipelines, and detailed information about the participants, recordings, and transcripts. Furthermore, we explain how researchers can access and navigate the corpus via Databrary.

## Methods

### Study design

This corpus is based on an observational study design which also included caregiver-report measures. Before the lab visit, caregivers completed questionnaires asking about their infants’ demographic backgrounds, families’ socioeconomic status, and infants’ vocabulary and pointing gesture development. During the lab visit, caregivers and infants read a “first words” picture-book. Caregivers were instructed to read with their infant how they typically would at home. The session was video recorded using an overview camera that captured a frontal view of the interaction, as well as two head-mounted cameras worn by both the caregiver and the infant, providing views from their individual perspectives. The session was also audio recorded using a microphone attached to the overview camera. The shared reading sessions had an average duration of 7.74 minutes, with a standard deviation of 3.02 minutes.

### Participants

The corpus included 44 English-speaking caregivers (41 mothers, 3 fathers) and infants (22 girls, 22 boys) with a mean age of 13.74 months (*SD* = 0.52, range = 13–14 months). Local families living in the Coventry and Warwickshire (UK) areas were recruited via the University of Warwick’s family database. Infants’ ethnicity was predominantly reported as White British/Irish (*n* = 38), with some Asian/Asian British (*n* = 1), North African (*n* = 1), and Mixed (*n* = 4). All infants were typically developing, with no known developmental disorders or delays reported by their caregivers. All caregivers reported that English was the primary language spoken in the home, with infants being exposed to English at least 70% of the time. Caregivers who indicated that other languages besides English were spoken in the home, also specified British Sign Language (*n* = 1), Catalan (*n* = 1), Cantonese (*n* = 1), Moroccan Arabic (*n* = 1), Polish (*n* = 2), Punjabi (*n* = 2), and Spanish (*n* = 1). The demographic information of the full sample is in Table 1. Caregivers were reimbursed for parking expenses for the families’ lab visit. Infants received a teddy bear and a “Junior Scientist Diploma” as a thank-you for participating.

**Table 1:** Demographic and Socioeconomic Sample Characteristics (N = 44).

Demographic Characteristics	Description	*N* (%)
Sex	Male	22 (50%)
	Female	22 (50%)
Age	13–month-olds	32 (72.7%)
	14–month-olds	12 (27.3%)
Ethnicity	White British/Irish	38 (86.3%)
	Asian/Asian British	1 (2.3%)
	Mixed Ethnicity (White and Other)	4 (9.1%)
	Other ethnic groups (Namely: North African)	1 (2.3%)
Caregiver’s relation to infant	Mother	41 (93.2%)
	Father	3 (6.8%)
Caregiver education	GCSE/O Level/NVQ Level 1 or 2/similar	3 (6.8%)
	A level/ NVQ Level 3/ similar	2 (4.5%)
	University degree/HND/ HNC/ NVQ Level 4 or 5/similar	20 (45.5%)
	Postgraduate/ similar e.g., (PGCE, PhD, MA etc.)	19 (43.2%)
Household income	£24,001–£42,000	4 (9.1%)
	£42,000 or more	38 (86.4%)
	Preferred not to disclose	2 (4.5%)

*Note*. ‘Caregiver’s relation to infant’ and ‘Caregiver education’ refers to the caregiver who participated in the study. For Ethnicity, Caregiver education, and Household income, more options were available, but none of the caregivers selected them.

The study obtained full ethical approval from the Psychology Department Research Ethics Committee at the University of Warwick (PSY_PGR_22-23/33). Caregivers provided informed written consent for their own participation and on behalf of their infant. In addition to consenting to take part in the study, caregivers were given the option to consent to share their survey responses and the audiovisual recordings of their shared book reading sessions via Databrary, which is a secure online repository for developmental research. Caregivers who provided this additional optional consent completed the full Databrary Release Form (https://databrary.org/support/irb/release-template), which explained that their shared recordings may include direct identifiers such as names spoken aloud, images, and voices which may be recognisable. The Databrary release form also specified that only authorised researchers, who have agreed to uphold strict confidentiality requirements, can access their data via Databrary. Caregivers were explicitly reminded that anything said or done during the session would be captured in the recordings and shared with authorised Databrary users. Caregivers were also informed precisely when audiovisual recordings began and ended.

An additional 8 caregiver-infant dyads participated in the study but were excluded from the corpus, because caregivers did not provide optional consent to share data via Databrary (*n* = 6), there were technical issues during the lab visit (*n* = 1, camera malfunction), or the family did not meet the language criteria (*n* = 1, English was not the primary language spoken in the home).

### Caregiver-report measures

We asked caregivers to complete three online questionnaires the day before the lab visit, covering infants’ demographic and socioeconomic information, as well as vocabulary and pointing gesture development.

#### UK-CDI family questionnaire

We collected socioeconomic and demographic data on infants using the UK-CDI Family Questionnaire^61^, selecting questions from Section A (1, 5, 6), Section B (4, 17, 23, 27), and Section C (4, 5, 6). These covered demographic details (e.g., child’s age, gender, ethnicity, caregiver income and education), language background (e.g., caregiver’s English fluency and infant’s language exposure), and developmental concerns (e.g., gestational age at birth, presence of developmental disabilities or sensory impairments).

#### UK-CDI words and gestures

We assessed infants’ vocabulary using the UK Communicative Development Inventory^61^, a normed caregiver-report measure. This checklist consists of 395 words and measures both infants’ comprehensive and productive vocabulary by asking caregivers to indicate for each word whether their infant either *understands and says* it, only *understands* it, or does not understand it. Words are categorised into groups such as sounds, animals, vehicles, and actions. An infant’s productive vocabulary is the total number of words the infant *understands and says*, while comprehensive vocabulary includes all words the infant *understands* and *understands and says*. We used the UK-CDI solely for vocabulary assessment and therefore did not use the actions and gestures section of the questionnaire, as we had a separate gesture measure.

#### QPOINT

Infants’ pointing gesture development was assessed using the QPOINT^62^, a caregiver-reported questionnaire. This tool evaluates two types of pointing: imperative pointing, where an infant points to make a request, and declarative pointing, where an infant points to share attention to something. Caregivers report how often their infant points in various scenarios. Examples of imperative pointing include pointing to a toy that is out of reach or pointing to bottle or food they want. Declarative pointing examples include pointing toward a sudden or unexpected noise or pointing to pictures in a book or magazine without touching them.

#### Picture-book

**Fig. 1:**
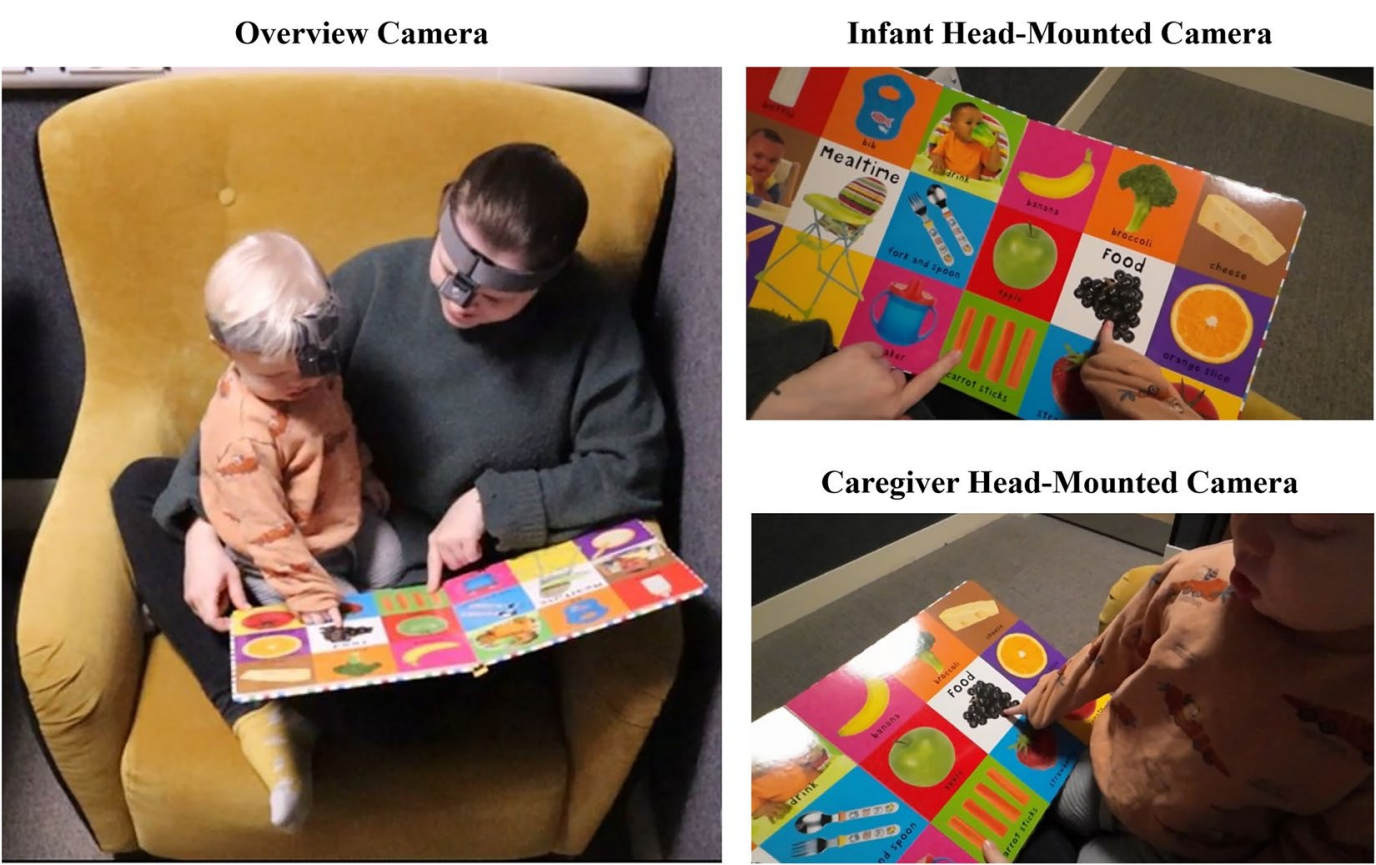
Examples of the Three Camera Views from the Reading Sessions. Note. Example of the three camera views captured during the reading sessions. The overview camera (left panel) captured the frontal view of the interaction. The infant’s head-mounted camera (top-right panel) recorded the first-person perspective of the infant, often capturing the caregiver’s hands, the infant’s hands, and the book. The caregiver’s head-mounted camera (bottom-right panel) recorded the first-person perspective of the caregiver, often capturing the infant, the infant’s hands, and the book. The caregiver depicted here provided explicit informed consent for both their own and their infant’s images to appear in scientific publications.

The picture-book read by all caregiver-infant dyads was a *First 100 Words* board book^74^. The book was suitable for infants aged 0-3 years. This book was selected to reflect the typical picture-books that may be read at home by families with 13–14-month-old infants. Many caregivers reported having read similar books at home, and 6 caregivers stated that they owned this exact book. Among those families, the number of times the book had been read at home ranged from never to 7 times. The book itself consisted of 14 pages with realistic pictures organised into categories such as mealtime, food, clothes, vehicles, and animals, with the category labels written in the centre of the page. Each page featured a colourful 3 × 3 grid, with each part of the grid showing a photo image and its corresponding English label written underneath it (see Fig. 1). A list of all the picture referents in the book is included in the corpus, located in the session folder *B) List of book referents*, as a CSV file titled *list_of_book_referents.csv*.

### Procedure

A step-by-step lab walkthrough video demonstrating the lab setup and study procedure is included in the corpus, located in the session folder *F) Lab walkthrough video*, as a video file titled *lab_walkthrough_video.mp4*.

Caregivers were asked to complete an online version of the UK-CDI family information questionnaire and vocabulary checklist^61^ and the QPOINT^62^ the day before the lab visit. Reading sessions took place in the Infant Lab at the University of Warwick. Upon arrival, families were welcomed by the researcher, who guided them to the reception area for a brief warm-up, where the infant engaged in free play with the researcher using age-appropriate toys (e.g., a shape sorter) to familiarise the infant with the researcher and the new environment. Caregivers chose to consent to sharing their data on Databrary via the Databrary Release form, which explained how their data might be shared or accessed. Providing permission for Databrary was optional. Next, the researcher instructed caregivers to fit head-mounted cameras on themselves and their infants using an elastic headband with adjustable Velcro straps. Caregivers first put on their own head-mounted camera, with the researcher ensuring correct positioning of the camera at the centre of the forehead. Caregivers then put on the head-mounted camera on their infants while the researcher distracted the infants by blowing bubbles using a bubble wand. Once the head-mounted cameras were fitted, the researcher guided the family to the sound-proof testing booth opposite the reception area. The testing area contained a reading chair, a small desk beside it, and an overview camera on a shelf with a microphone attached to it positioned opposite the reading chair for a frontal view of the reading session (see left panel of Fig. 1). Caregivers and infants positioned themselves comfortably in the reading chair and were given the *First 100 Words* book to read^74^. Caregivers were instructed to read with their infant naturally; in the same way they would usually read a book at home. The researcher then started the recordings, left the testing booth, and monitored the session via a live feed of the overview camera. Caregivers and infants read for as long as they wished, with an average reading session lasting approximately 8 minutes. Caregivers raised their hand in the air when they finished, and the researcher entered the testing area to stop the recordings.

### Audio-visual recording equipment

The reading sessions were recorded using high-quality audiovisual recording equipment. A Canon EOS 250D Digital SLR camera with an 18–55 mm IS STM lens was positioned as an overview camera to capture a frontal view of the reading session. Attached to the overview camera was a Rode VideoMic Pro R microphone with a deadcat sleeve for capturing high-quality audio recordings. To capture first-person perspectives, both caregivers and infants wore DJI Action 2 cameras, secured using DJI Action 2 Magnetic Headbands (see Fig. 1). The infant’s headband was customised by adding more Velcro straps to ensure a secure and adjustable fit for smaller head sizes.

### Caregiver speech transcription

Caregivers’ speech was automatically transcribed using WhisperX-based Python code^75^ with the large-v3 language model and WAV2VEC2_ASR_LARGE_LV60K_960H for alignment. The process was run locally on a GPU-enabled machine with a batch size of 16. To facilitate automatic transcription, we used Spleeter (2-stem model) to separate the vocal track from background accompaniment (e.g., environmental noise). Both the ‘vocals’ and ‘accompaniment’ stems were used in subsequent transcription^76^. The transcripts were based on the video recordings from the overview camera (.mp4). This auto-transcription procedure resulted in a single text file (.csv) for each recording, with time-stamped utterances for the caregiver on each row. A researcher (the first author) and research assistant then manually redacted infants’ names from the caregiver speech transcripts by replacing the mention of the name with <*name>* in the transcript, following standard practices in documenting and sharing child corpora^77^.

## Data Record

### Data overview

The Shared Book Reading Corpus is available at https://databrary.org/volume/1844^51^. The corpus comprises high-quality audiovisual recordings of 44 British English-speaking caregiver–infant dyads, each participating in a naturalistic shared reading session using the *First 100 Words* picture book^74^, conducted in a research lab. For each dyad, the corpus includes an overview camera recording, a caregiver head-mounted camera recording, and, in most cases, an infant head-mounted camera recording. In addition to the video data, the corpus contains for all 44 dyads, detailed transcriptions of caregiver speech from the reading sessions, demographic and socioeconomic information for all dyads, and caregiver-reported assessments of all infants’ vocabulary size and pointing gesture development, collected the day before the reading session. A corpus inventory file is included in the dataset, located in the session folder *A) Corpus Inventory*, as a CSV file titled *corpus_inventory.csv*. This file lists all available data files for each participating dyad. For each file type, availability is indicated using binary values: *TRUE* denotes that the file is available for a given dyad, while *FALSE* indicates it is not. A notes column is also included to flag any relevant issues or anomalies associated with specific files. If no issues were identified for a dyad’s files, this field is marked *NA*.

### Data file organisation and naming structure

Each dyad in the corpus was assigned a unique 8-character identifier as the *PARTICIPANTID* (e.g., *11APBREZ*), which was used consistently across all associated files. The data were organised with a session folder structure, where each dyad had a dedicated folder labelled *Shared book reading session* and was linked with their *PARTICIPANTID*. This folder contains the video and audio recordings from the shared book reading session, as well as the transcript of the caregiver’s speech. Video recordings were named according to the camera perspective: *PARTICIPANTID_video_overview.mp4* refers to the overview camera recording of the session, *PARTICIPANTID_video_caregiver.mp4* to the recording from the caregiver’s head-mounted camera, and *PARTICIPANTID_video_infant.mp4* to the recording from the infant’s head-mounted camera, where available. The file *PARTICIPANTID_audio.wav* contains the audio track extracted from the overview camera recording, and *PARTICIPANTID_transcript_caregiver_speech.csv* contains a full transcription of the caregiver’s speech, based on the overview camera recording.

Questionnaire data were stored separately from the shared book reading session folders, in three standalone session folders that each contained responses from all 44 dyads. Session folder *C) UK-CDI Family questionnaire responses*, contains the file *uk-cdi_family_responses.csv*, which includes responses to the UK-CDI Family Questionnaire^61^, providing demographic and socioeconomic information for each infant. Session folder *D) UK-CDI Words and Gestures questionnaire responses*, contains the file *uk-cdi_words_and_gestures_responses.csv*, which includes responses to the UK-CDI Words and Gestures Questionnaire^61^, assessing infants’ receptive and expressive vocabulary. Session folder *E) QPOINT questionnaire responses*, contains the file *qpoint_responses.csv*, which includes responses to the QPOINT Questionnaire^62^, capturing information about infants’ pointing gesture development.

## Technical Validation

The caregiver speech transcripts in our corpus are 100% accurate. After the auto-transcription procedure, the text files were manually checked by a researcher (the first author) and a research assistant. The researchers reviewed the recordings of the overview camera alongside the transcribed text, correcting any mistakes (e.g., when the word *“pear”* was automatically transcribed as *“pair”*), and removing infant vocalisations (e.g., babble noise).

A researcher (the first author) also reviewed all survey response data as well as all video and audio files to assess their availability and quality for each dyad’s reading session. All survey responses, overview camera recordings, and caregiver head-mounted camera recordings were available for the 44 sessions. Infant head-mounted camera recordings were available for 29 of the 44 sessions. In the remaining 15 sessions, infants refused to wear the head-mounted camera, and thus only the caregiver and overview perspectives were available. Of the 29 sessions where infants initially wore the camera, in 12 cases the infants kept it on for the entire session, while in the other 17 cases it was removed part-way through and placed on a nearby desk, where it occasionally continued to capture a side-view of the interaction.

One technical issue was identified for one dyad (Participant ID: 8CNKE7RV), where both the overview camera and infant head-mounted camera stopped recording before the end of the session. The audio file included in the corpus is from the high-quality microphone that was attached to the overview camera. Therefore, the high-quality audio file also ends earlier than the reading session. However, the caregiver head-mounted camera, which had a built-in microphone, captured the full session. As a result, the end of the reading session was still captured, although it can only be viewed and heard from the caregiver’s perspective, with slightly lower audio quality.

## Usage Notes

### Repository storage and access information

The corpus was documented on Databrary and can be accessed at https://databrary.org/volume/1844^51^. Access to the dataset is controlled and requires prospective users to apply for access via the Databrary platform (https://databrary.org). Access to the corpus is restricted to principal investigators and affiliated researchers at institutions that have signed a Databrary access agreement, as well as to authorized organizational representatives. To protect the privacy of research participants, all Databrary users must be affiliated with an institution that requires researchers to complete training in ethical guidelines and procedures for human subjects as well as obtain approval for new research via an ethics review board.

Researchers interested in accessing the dataset must first create a Databrary account and initiate the authorisation process. Principal investigators must be vetted by Databrary staff and provide documentation of their institution’s ethics training and review procedures. Once authorised, they may also sponsor affiliated researchers (e.g., students or staff) who will be granted access under their supervision. Institutions must also sign a Databrary Access Agreement, which outlines data privacy responsibilities.

The dataset associated with this study is shared under Databrary’s “Authorized Users” level of access. This means that identifiable data can be accessed only by authorised investigators who agree to uphold Databrary’s terms of use. There are no additional manual access controls imposed by the authors beyond those managed by the Databrary platform.

A publicly accessible file titled *repository_contents.txt*, which summarises the full contents of the dataset, is available in the Databrary repository. It is shared under Databrary’s “Public” access level, meaning it can be viewed by anyone without requiring Databrary registration or authorisation. This file is intended to provide prospective users with a clear overview of the types of data available before initiating the Databrary access application process.

### Limitations

While the Shared Book Reading Corpus^51^ offers rich, multimodal data from 44 caregiver–infant dyads, several limitations should be acknowledged. First, the sample is relatively homogenous in terms of demographic background: most participating families identified as White, middle-class, and highly educated. As such, the behaviours captured in this corpus may not fully reflect the diversity of caregiver–infant interactions across broader cultural, socioeconomic, and racial or ethnic groups. Users of the corpus should be cautious when generalising findings beyond populations similar to those represented here.

Second, the data were collected in a semi-naturalistic laboratory setting which may have influenced participants’ behaviour. For example, caregivers may have been aware that a researcher was observing them, and infants may have been sensitive to the new environment or wearable camera equipment. While the controlled lab setting helps ensure consistent, high-quality recordings (in a sound-attenuated testing area) and supports comparability across sessions (through offering the same picture-book in the same reading setting), it may reduce the ecological validity of the interactions compared to reading sessions captured in the family home. To help address this limitation, caregivers were instructed to read naturally with their infant as they would at home: they received no specific instructions, used a commercially available first-word picture book, sat in a comfortable reading chair, and completed the session without the researcher physically present in the room, creating a semi-naturalistic setting.

Third, infant head-mounted camera recordings are only available for a subset of the corpus (29 sessions of 44). In 15 sessions, infants refused to wear the head-mounted camera, which may introduce bias. For example, it is possible that infants who declined to wear the head-mounted camera shared particular traits, such as heightened sensitivity to sensory stimuli, lower tolerance for unfamiliar environments, or higher temperamental reactivity. This could result in systematic differences between the reading sessions with and without infant-perspective data. Additionally, even among sessions with infant head-mounted camera footage, some infants removed the camera partway through the session, leading to only partially analysable recordings. As such, researchers using the dataset should account for the presence or absence of infant head-camera data in their analyses and be cautious when comparing across dyads with differing camera availability. However, we would like to note that for researchers who are interested in head-mounted camera data for the purpose of analysing book-related behaviours up-close, the caregiver head-mounted camera recordings are available for all 44 dyads.

## Data Availability

The code to generate automatic time-stamped transcription from the overview video camera recordings, which we used for caregiver speech, is available via GitHub (link).
